# A high-gain dual-band shared-aperture array integrating zero-order-resonance patch and higher-order metasurface antenna

**DOI:** 10.1038/s41598-023-38562-3

**Published:** 2023-07-19

**Authors:** Qian Chen, You-Feng Cheng, Chen-Hao Shao, Ju Feng, Cheng Liao, Xiao Ding

**Affiliations:** 1 Institute of Electromagnetics, Southwest Jiaotong University (SWJTU), Chengdu, 610031 China; 2 Institute of Applied Physics, University of Electronic Science and Technology of China (UESTC), Chengdu, 610054 China

**Keywords:** Electrical and electronic engineering, Engineering

## Abstract

This letter proposes a high-gain shared-aperture array for vehicular communications which integrates the hybrid zeroth-order-resonance (ZOR) patch and the higher-order metasurface antenna (MA). In terms of each array element, four hybrid ZOR patches with shorting pins form a metasurface which is fed by slot coupling. Different from some reported designs which have more high-frequency elements than low-frequency ones, the whole array is composed of 4 $\times$ 4 hybrid ZOR patches operating in *C* band (4.74–5.12 GHz) and 2 $\times$ 2 MAs working in *Ku* band (13.0–14.0 GHz). Such a layout brings that the designed array has the benefit to reduce *Ku*-band loss. Measure results validate the dual-band operation and show that the antenna has its peak gains of 12.7 dBi and 17.2 dBi in these two bands, respectively, which can support vehicle-to-base station and vehicle-to-satellite communications.

## Introduction

Due to its high aperture-utilization efficiency, the shared-aperture antenna has drawn considerable attention^1^ The concept adopted in shared-aperture antennas is to integrate dual/multiple antennas with diversities in terms of the frequency^2^, radiation^3^, and polarization^4^ into a single structure. Among them, shared-aperture antennas with diverse operation bands (especially those with the large frequency ratio) have broad applications, such as the wireless communication system^5^, the vehicular communication^6^, the low earth orbit satellite communication system^7^, and the system integration of terrestrial mobile communication and satellite communication^8^. In addition, shared-aperture arrays with the large frequency ratio and high realized gains have ignited intensive attentions in the past decade. In terms of the dual-band shared-aperture array, many solutions have been reported in literature, such as the stacked designs^9,10^, the co-planar designs^11,12^, and the shared-structure designs^13–15^. The last one has advantages of low profile, high structure reuse ratio, and the avoidance of the block effect.

Due to the different physical dimensions of the dual-band elements in a shared-aperture array, generally it has more high-frequency (HF) elements than low-frequency (LF) ones. For instance, a dual-band shared-aperture antenna composed of 4 $\times$ 4 *Ku*-band patches and 2 $\times$ 2 *C*-band slots was presented in^16^. However, it is well known that, when the HF array operates in the frequency band above *C* band, the propagation loss is quite huge. That brings significant losses in the design of the HF feeding structure. In^16^, in order to reduce the loss in *Ku* band, a complicated feeding network is designed for the *Ku*-band array to realize the high broadside gain. Therefore, the element number of the HF array should be suppressed in the design of a dual-band shared-aperture array, while the aperture efficiency and realized gain should be maintained.

To this hence, a novel high-gain, dual-band, shared-aperture array, which consists of 4 $\times$ 4 *C*-band hybrid zeroth-order-resonance (ZOR) patches and 2 $\times$ 2 *Ku*-band higher-order metasurface antennas (MAs), is presented and analyzed in this letter. The proposed array has a shared-structure design with a high structure reuse ratio. The radiation shorted patches of the hybrid ZOR array form the metasuface of the MA array. The metasurface is simultaneously used as the radiator in both bands. In the low-frequency band, the metasuface is adopted as an array composed of four patches. Since the patches works in the hybrid ZOR mode, the radiation aperture can be reduced sufficiently. In the high-frequency band, the metasuface antenna operates based on higher-order modes, which makes the radiation aperture been enlarged. Therefore, when the two antennas are integrated as an array, the element number in the high-frequency band can be reduced to less than that in the low-frequency band. When the hybrid ZOR patch array works, it has an impedance bandwidth of 4.74 $\sim$ 5.12 GHz and a realized peak gain of 12.7 dBi. The higher-order MA array operates from 13.0 to 14.0 GHz and has its peak gain of 17.2 dBi in this band. The proposed array also shows good isolation between two ports.

Different from reported shared-aperture antennas, this design has more LF elements than HF ones. Such a design has the following two advantages. First, the element number of HF array is significantly suppressed compared to some reported designs, which has benefit to decrease the HF propagation loss. Second, this structure-reused design maintains good efficiency and realized gain both in LF and HF band. Besides, due to the reduction in the number of high-frequency elements, the feeding structure is simplified, and the profile of the antenna is also decreased.

**Figure 1:**
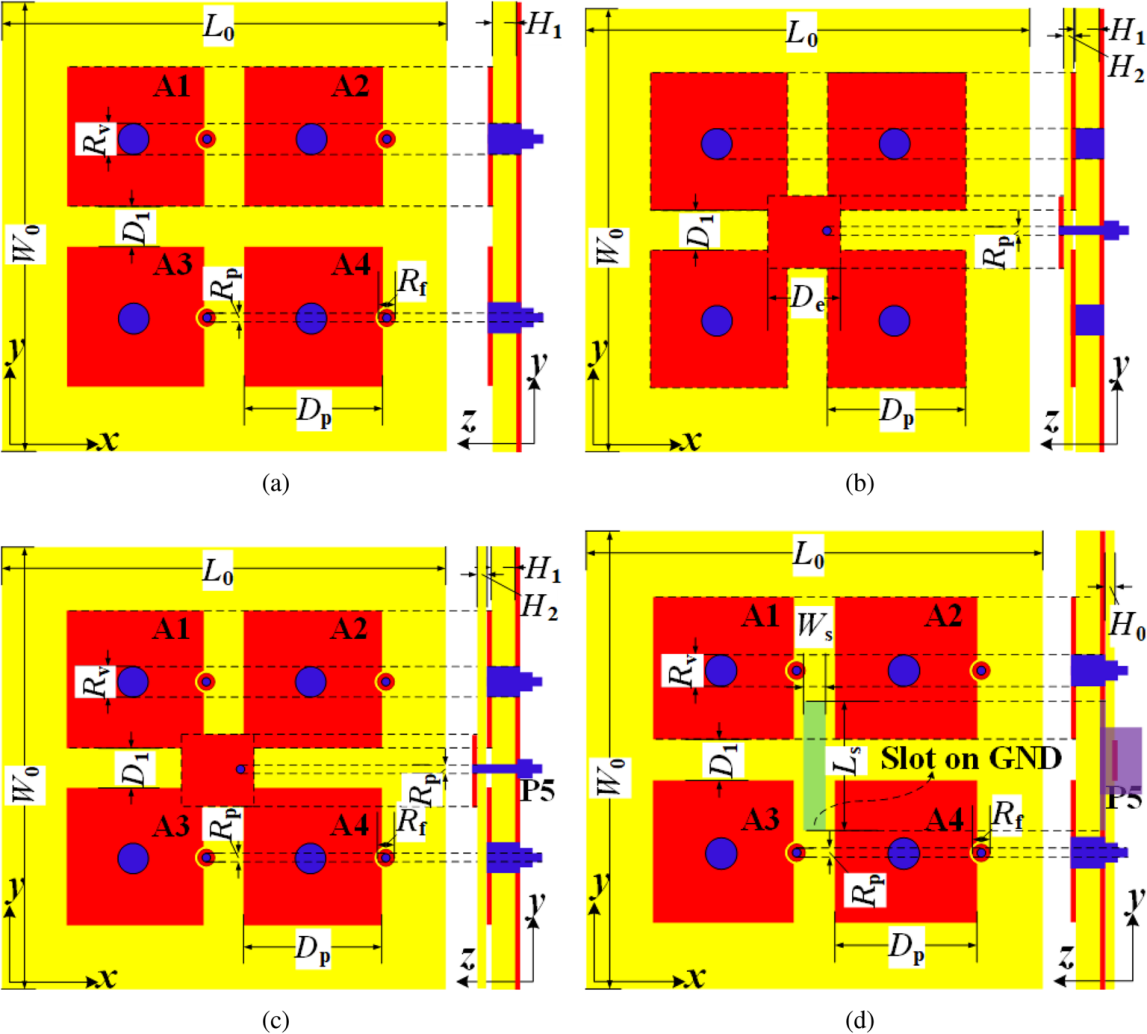
Geometries of initial antennas. (**a**) 2$\times$2 hybrid ZOR patch array. (**b**) ME antenna. (**c**) IE1. (**d**) IE2.

**Figure 2:**
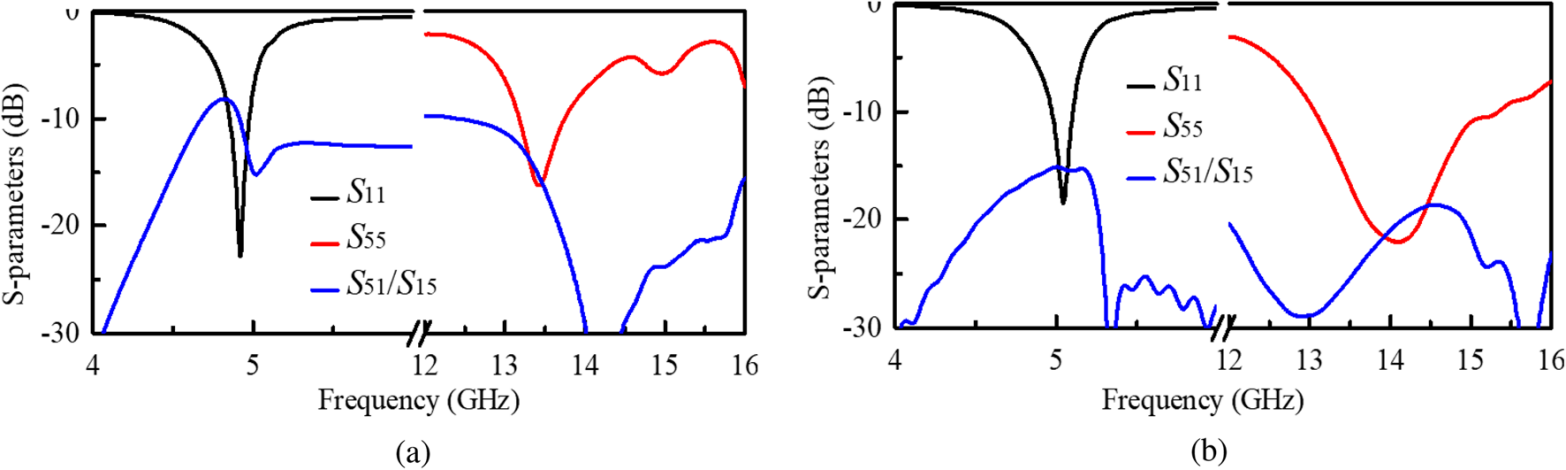
Simulated S-parameters of initial elements. (**a**) IE1. (**b**)IE2.

## Element design and analysis

Initially, a 2$\times$2 hybrid zeroth-order-resonance (ZOR) patch array is evolved from the designs in^17^ and^18^ and shown in Fig.1a. Each element of the array has a mushroom-shaped patch and is fed via capacitive coupling. It can also be observed that the mushroom-shaped patch array is similar to the radiation structure of the MA. Inspired by this, an MA fed by a printed patch is designed and plotted in Fig. 1b. Another thin substrate is placed above the mushroom-shaped patch array to support the feeding strips. The above two kinds of antennas resonate at *C* and *Ku* bands, respectively.

By integrating the two designs, as depicted in Fig. 1c, an initial element (IE1) antenna with the shared mushroom-shaped structure is realized. The MA is fed by port P5 and denoted as A5. Fig. 2a presents the simulated S-parameters of A1 and A5. It is seen that A1 and A5 resonate at 4.9 and 13.5 GHz, respectively. However, the operation bandwidth is quite narrow, and the isolations between the two ports within the operation bands are poor.

In order to enhance the reflection and isolation performance, the aperture-coupled excitation is introduced and applied to the MA^19^. As shown in Fig. 1d, a rectangle slot with its length of $L_{\mathrm{{s}}}$ and width of $W_{\mathrm{{s}}}$ is etched on the ground, and a feeding strip is printed on the additional bottom substrate. Likewise, the MA is denoted as A5 and fed by P5. In this situation, the *S*-parameters of A1 and A5 of the Initial element (IE2) are drawn in Fig. 2b. It is observed that the bandwidth of A5 is significantly improved from 0.5 to 2.3 GHz. Besides, the isolations in dual bands are also enhanced (both higher than 15 dB).

**Figure 3:**
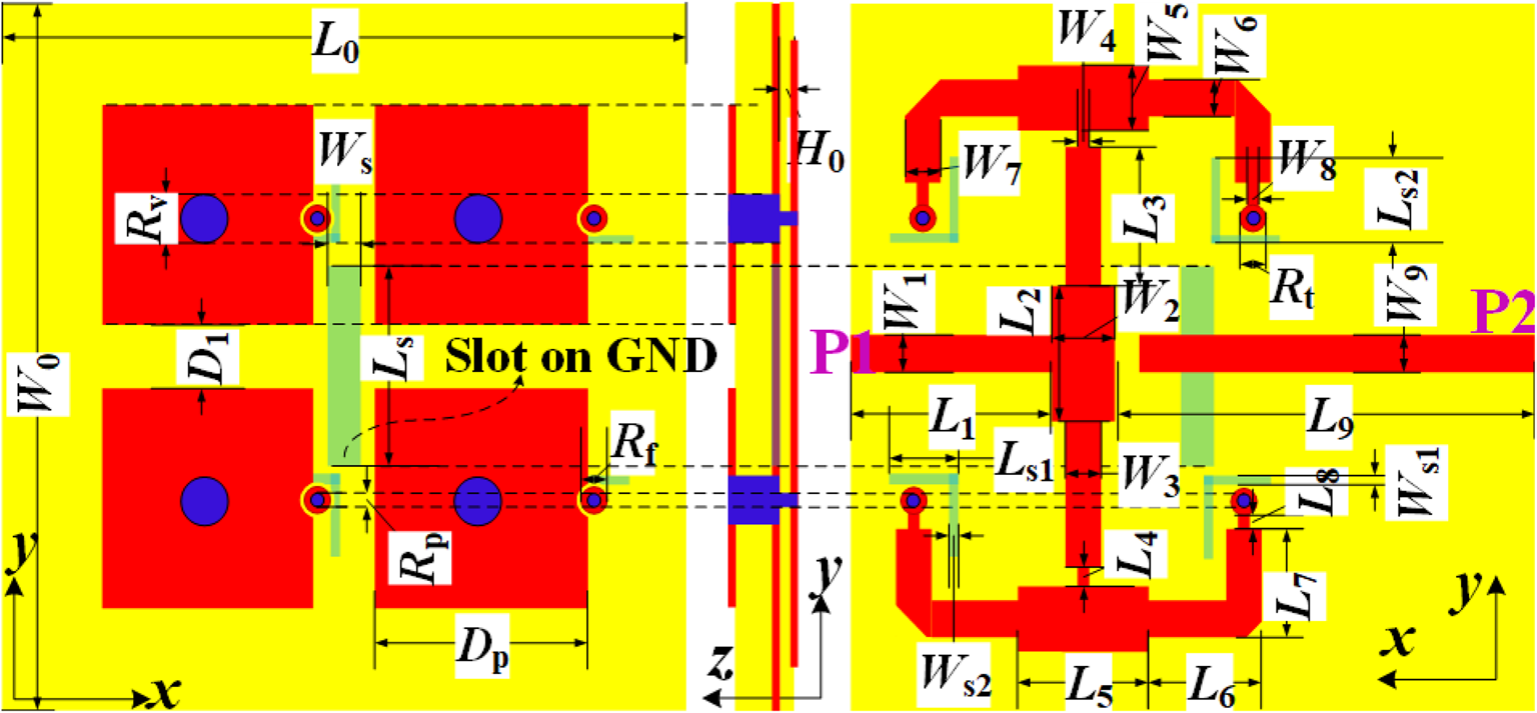
Geometry of the improved element antenna.

Then, A1 $\sim$ A4 shown in Fig. 1d are integrated by designing a feeding network and the final element antenna is obtained as shown in Fig. 3. In this case, the hybrid ZOR patch array and the MA are denoted as A1 and A2, respectively. Four metal pins penetrate the upper and lower substrates and connect the top patches and the bottom feeding network. Besides, there exists a circular slot etched on the ground at the position of each pin passing through the ground. Note that, Four L-shaped slots are etched on the ground to reduce the coupling between A1 and A2.

**Figure 4:**
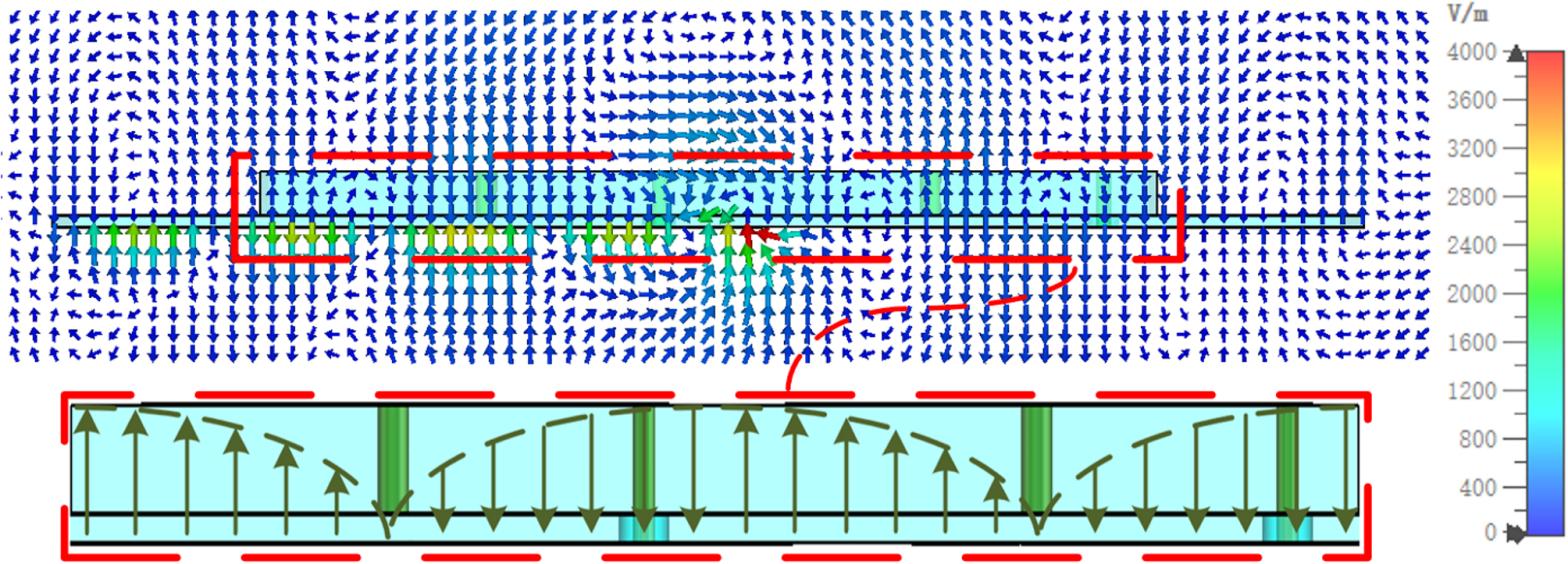
Simulated E-field distribution on *y*=0 plane of the MA at 13.5 GHz.

Fig. 4 plots the simulated E-field distribution of A2. It is observed that, the E-field distribution above the metasurface is similar to the TE$_{\mathrm{{20}}}$ mode of a conventional patch antenna at 13.5 GHz. The excited out-of-phase components $E_{\mathrm{{z}}}$ in the central region ensure the in-phase radiation field distributions along the metasurface and open edges. That is the reason why the *Ku*-band MA has a much larger aperture than the *C*-band hybrid ZOR patch.

**Figure 5:**
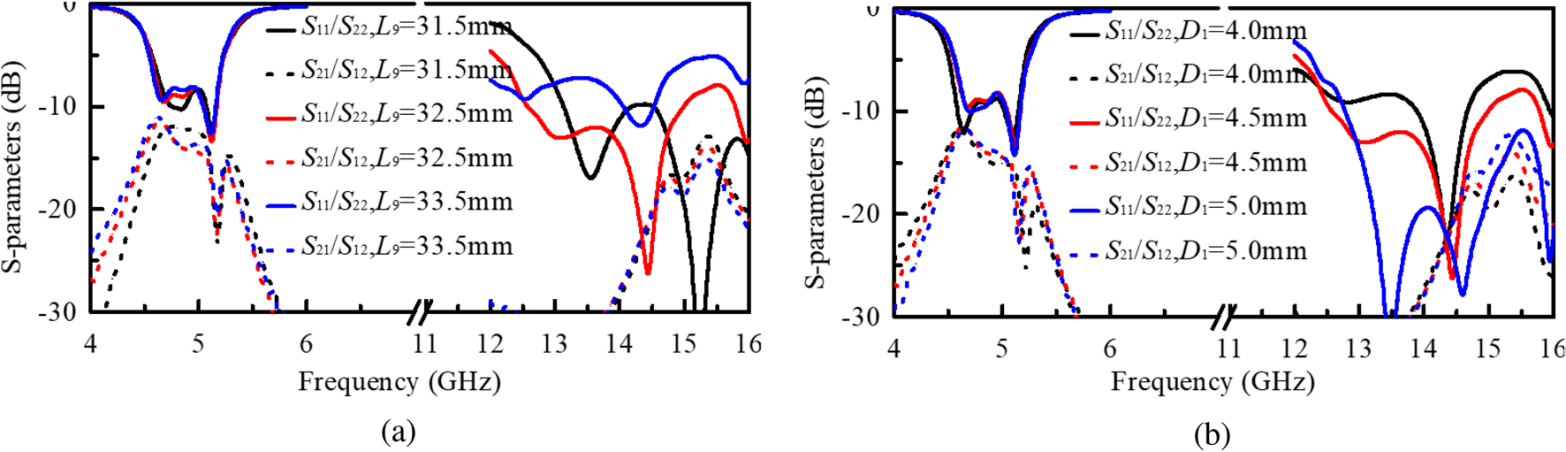
Effects of $L_{\mathrm{{9}}}$ and $D_{\mathrm{{1}}}$ on S-parameters. (**a**) $L_{\mathrm{{9}}}$. (**b**) $D_{\mathrm{{1}}}$.

In order to figure out the played roles of the feeding structure and the antenna dimension, here some comparisons and parametric studies are carried out. The influence of the length ($L_{\mathrm{{9}}}$) of the feeding line of A2 on the reflection and isolation performance is firstly investigated. As plotted in Fig. 5a, it is seen that $L_{\mathrm{{9}}}$ has some effects on dual resonances of A2. Besides, when $L_{\mathrm{{9}}}$ increases, the isolation between the two ports is enhanced, especially in the lower band. $L_{\mathrm{{9}}}$ is finally chosen as 32.5 mm to realize a broad bandwidth of A2. In addition, compared with the bandwidth of A1 shown in Fig. 2, the impedance bandwidth is broadened when the feeding network is used for the hybrid ZOR patch array.

Secondly, simulated reflection and transmission coefficients with different distances ($D_{\mathrm{{1}}}$) between these hybrid ZOR patches are studied and plotted in Fig. 5b. It is observed that $D_{\mathrm{{1}}}$ also has little impacts on the reflection performance of A1 but great impacts on the bandwidth of A2. With the increase of $D_{\mathrm{{1}}}$, the impedance matching of the lower resonance of A2 gets better and thus the bandwidth gets broadened, and the isolation in the higher band get worse. Therefore, $D_{\mathrm{{1}}}$ is selected as 4.5 mm.

Detailed physical parameters of the these element antennas are: $D_{\mathrm{{1}}}$ = 4.5 mm, $D_{\mathrm{{e}}}$ = 5.0 mm, $D_{\mathrm{{p}}}$ = 16.0 mm, $H_{\mathrm{{0}}}$ = $H_{\mathrm{{2}}}$ = 0.508 mm, $H_{\mathrm{{1}}}$ = 2.0 mm, $L_{\mathrm{{0}}}$ = $W_{\mathrm{{0}}}$ = 60 mm, $L_{\mathrm{{1}}}$ = 20.25 mm, $L_{\mathrm{{2}}}$ = $L_{\mathrm{{5}}}$ = 10.0 mm, $L_{\mathrm{{3}}}$ = 8.8 mm, $L_{\mathrm{{4}}}$ = 1.25 mm, $L_{\mathrm{{6}}}$ = 6.35 mm, $L_{\mathrm{{7}}}$ = 3.0 mm, $L_{\mathrm{{8}}}$ = 3.6 mm, $L_{\mathrm{{9}}}$ = 32.5 mm, $L_{\mathrm{{s}}}$ = 16.0 mm, $L_{\mathrm{{s1}}}$ = $L_{\mathrm{{s2}}}$ = 6.5 mm, $R_{\mathrm{{f}}}$ = 0.8 mm, $R_{\mathrm{{p}}}$ = 0.35 mm, $R_{\mathrm{{t}}}$ = 1.25 mm, $R_{\mathrm{{v}}}$ = 0.5 mm, $W_{\mathrm{{1}}}$ = $W_{\mathrm{{4}}}$ = $W_{\mathrm{{8}}}$ = $W_{\mathrm{{9}}}$ =1.3 mm, $W_{\mathrm{{2}}}$ = $W_{\mathrm{{5}}}$ = 3.5 mm, $W_{\mathrm{{3}}}$ = $W_{\mathrm{{6}}}$ = $W_{\mathrm{{7}}}$ = 2.2 mm, $W_{\mathrm{{s}}}$ = 2.0 mm, and $W_{\mathrm{{s1}}}$ = $W_{\mathrm{{s2}}}$ = 0.6 mm. All the substrates are F4BM with the relative permittivity of 2.65.

**Figure 6:**
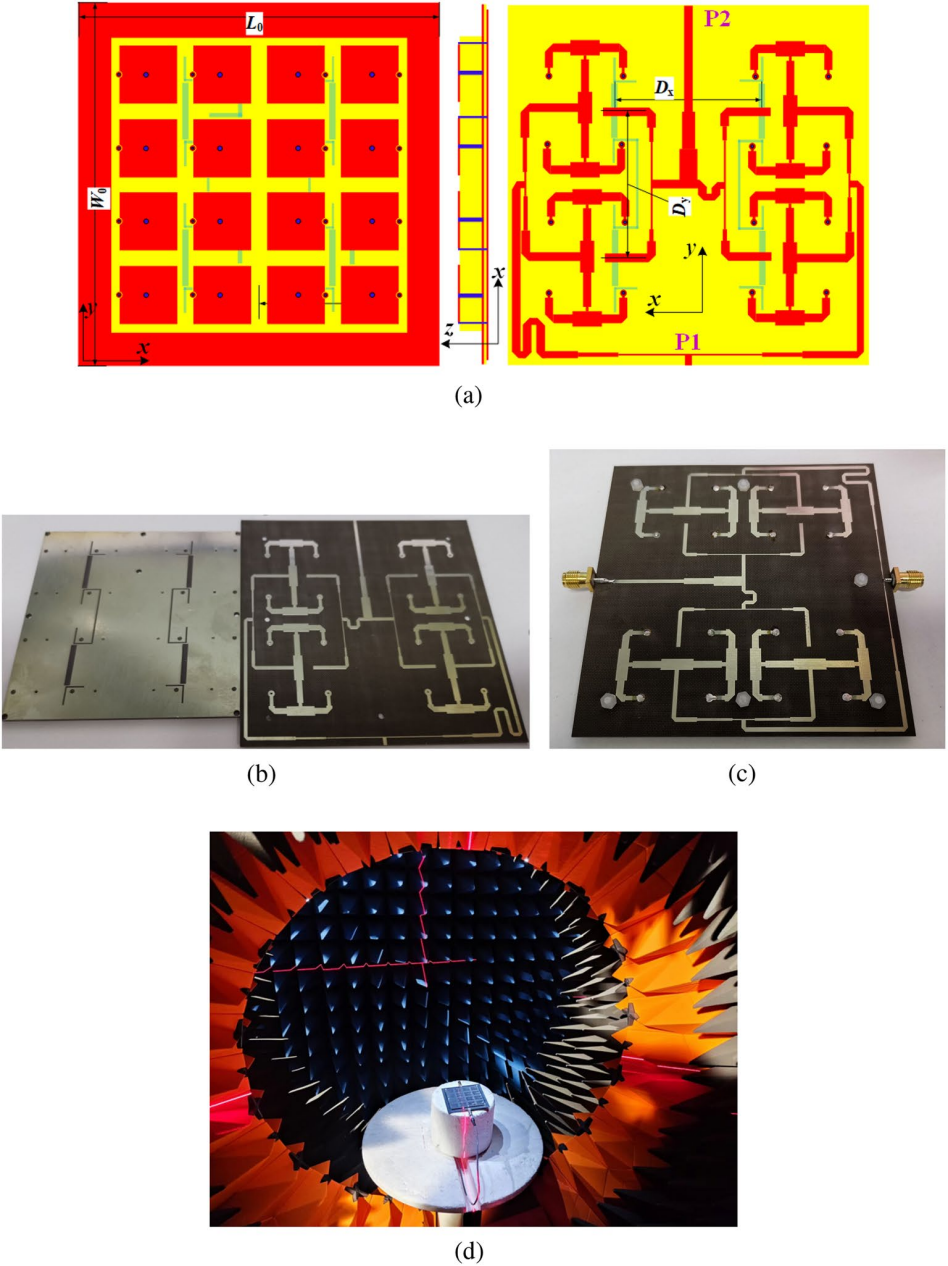
Geometry and fabricated prototype of the final array antenna. (**a**) Structure. (**b**) Antenna components before assembly. (**c**) Assembled prototype. (**d**) Measurement environment.

**Figure 7:**
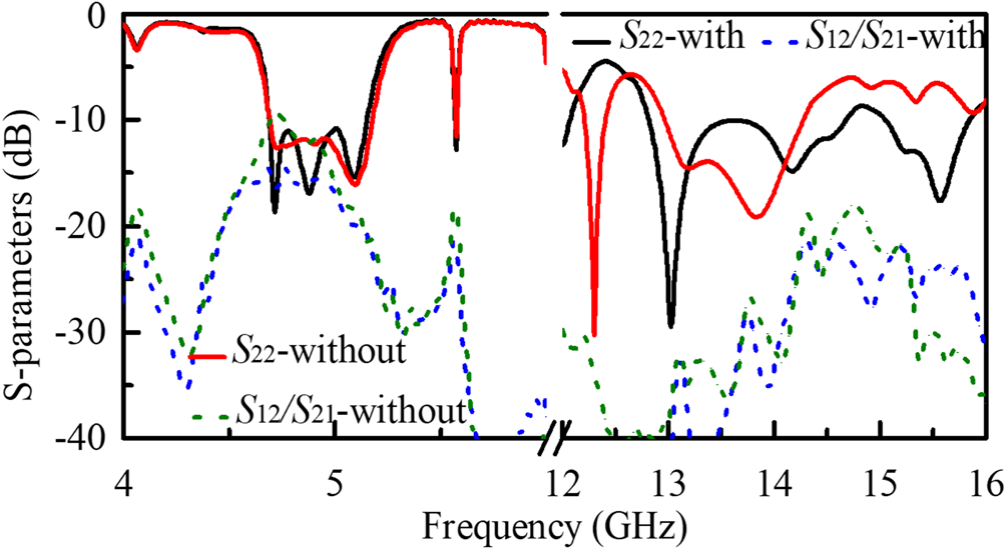
Simulated S-parameters of the array antennas with and without the split-ring shaped and L-shaped slots.

## 2$\times$2 array and final performance

Based on the above improved element, a 2$\times$2 shared-aperture array antenna is developed. As plotted in Fig. 6a, 4$\times$4 mushroom-shaped patches form the radiation aperture which corresponds to 16 hybrid ZOR patch elements and 2$\times$2 MA elements. The inter-element distances between the MA elements are chosen as $D_{x}$ = $D_{y}$ = 41.0 mm. All these elements are arranged periodically. The feeding structure, which is designed for the upper and lower frequency bands independently, has a 16-way and a four-way power dividers applied to the hybrid ZOR patch array and MA array, respectively. Note that, two symmetrical split-ring shaped and four L-shaped slots are etched on the ground plane to reduce the coupling between the two arrays. Physical parameters of the antenna structure and electromagnetic parameters of these adopted substrates are all the same as those of the above final element.

The array antenna is fabricated and measured. Measured results are compared with simulated ones to validate the reflection and radiation performance. The array components before assembly and the assembled prototype are illustrated in Fig. 6b and c. The array is measured in a SATIMO anechoic chamber as shown in Fig. 6d. When port P1 is excited and port P2 is terminated in matched loads, the hybrid ZOR patch array works and radiates broadside patterns in *C* band. On the contrary, when port P2 is only excited, the *Ku*-band MA array operates and also generates the broadside radiation.

In order to investigate the isolation enhancement of the split-ring shaped and L-shaped slots, Fig. 7 shows the simulated results and some comparisons. It is observed that the loading of these slots has little effects on the reflection characteristics of Port 1. The isolation between Ports 1 and 2 is improved from 9.5 to 15.1 dB in the LF band when these slots are loaded and it is also enhanced by about 3 dB in the HF band. Besides, the reflection performance of Port 2, such as the impedance bandwidth, gets improved significantly. It can be summarized that the etched slots can improve the reflection features in the HF band and reduce the mutual coupling between the two ports in both low and high-frequency bands.

**Figure 8:**
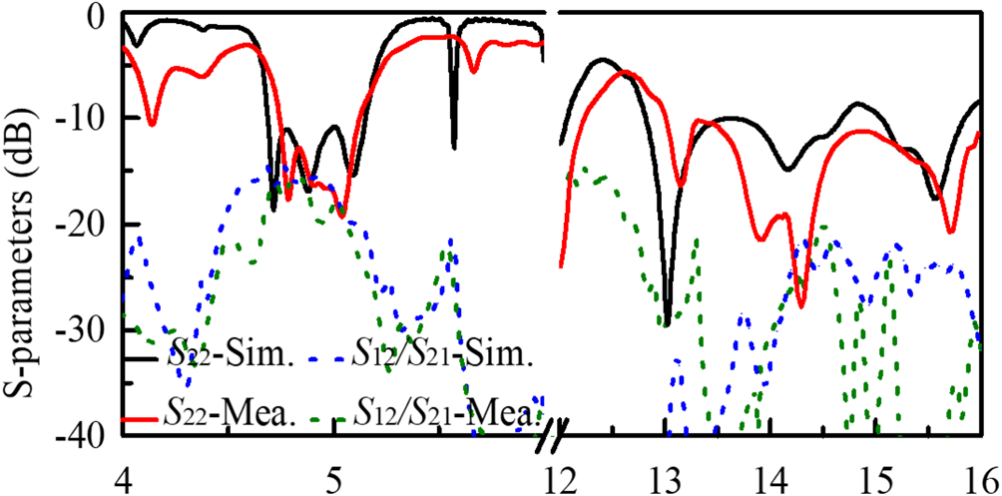
Simulated and measured *S*-parameters of the final array antenna.

**Figure 9:**
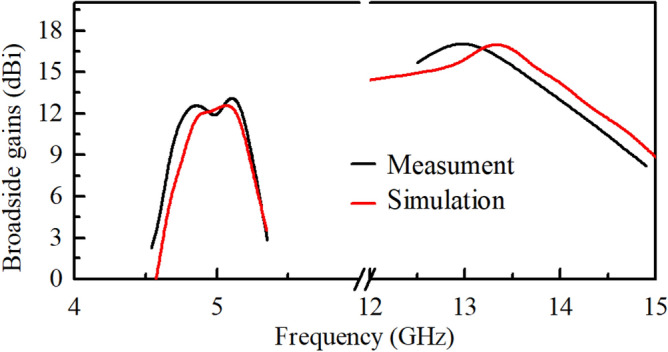
Simulated and measured peak gains of the final array antenna.

**Figure 10:**
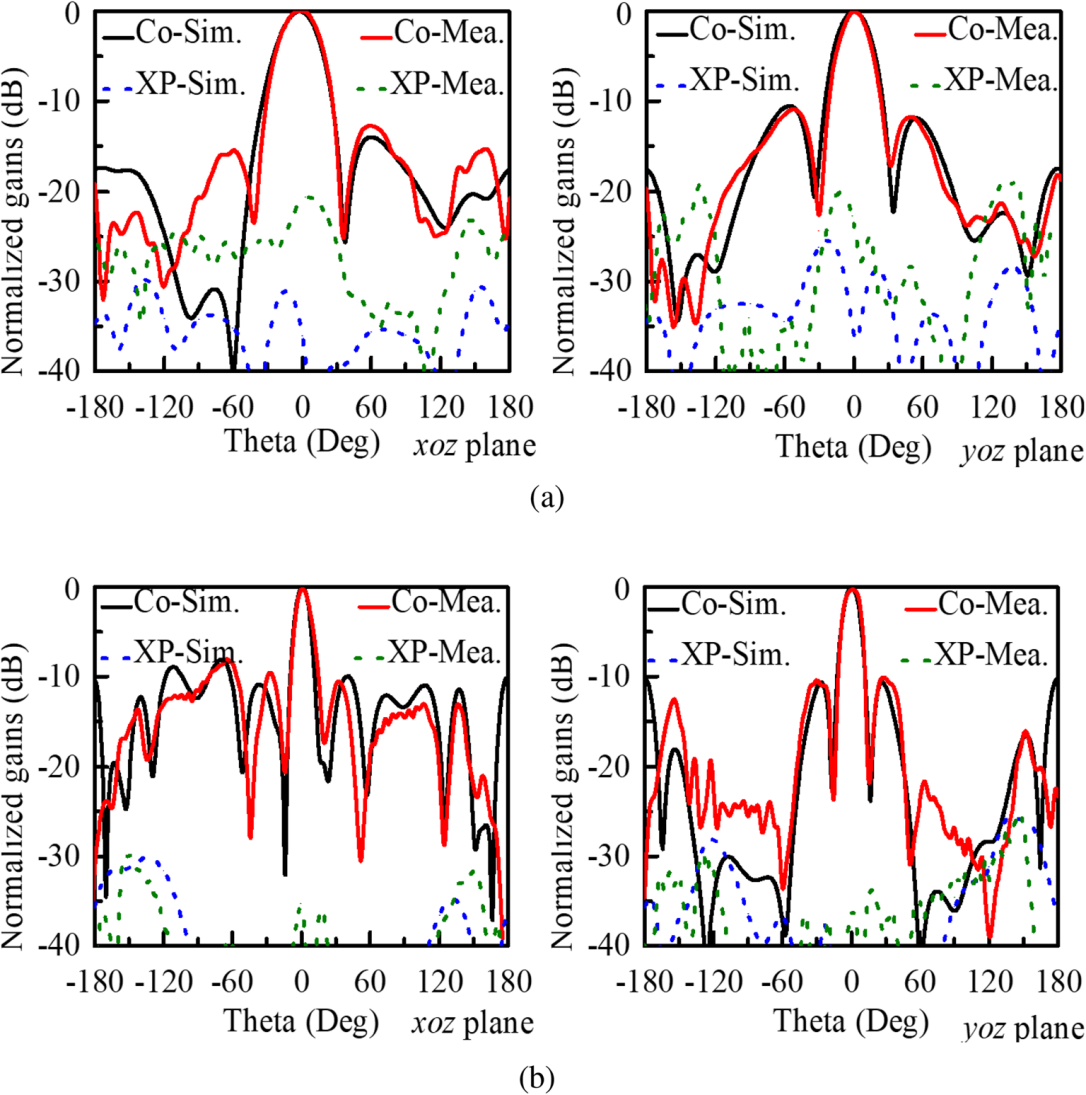
Simulated and measured peak gains of the final array antenna. (**a**) 5.0 GHz. (**b**) 13.5 GHz.

The measured reflection and radiation characteristics of the final array antenna are presented and compared with simulated ones. Fig. 8 shows the reflection and transmission coefficients in the low- and high-frequency band. The measured -10-dB reflection bandwidth of Port 1 is 4.74 $\sim$ 5.12 GHz (7.1$\%$) which is a little narrower than the simulated one (4.69 $\sim$ 5.15 GHz). Besides, in this band, both the simulated and measured isolations between Ports 1 and 2 are higher than 15 dB. In term of the HF band, it can also be found that Port 2 has the measured reflection lower than -10 dB in the frequency range of 13.0 $\sim$ 16.0 GHz. Compared to the simulated results, the resonant frequency band is shifted upward to the HF band. It is worthwhile to be noted that, the antenna cannot maintain good radiation in the whole impedance band, and the gain bandwidth would be analyzed below. It can also be seen that the measured isolation between the two ports is higher than 21.0 dB in this band.

In terms of the radiation performance, the realized broadside gains are drawn in Fig. 9. In the LF band, the peak gain is 12.7 dBi which appears at 5.05 GHz, and the 3-dB gain bandwidth is about 4.80 $\sim$ 5.20 GHz which agrees well with the impedance bandwidth. In terms of the realized gains in the HF band, it is observed that the measured results also have a frequency shift upward to the HF band compared with the simulated ones. Besides, as plotted in Fig. 9, the 3-dB gain bandwidth is about 12.5 $\sim$ 14.0 GHz, and the realized peak gain is 17.2 dBi (at 13.25 GHz) which is a little lower than the simulated one (17.8 dBi).

Figure 10 presents the normalized gain patterns of the array antenna in the *xoz* and *yoz* planes at different frequencies. Measured results agree well with simulated ones, especially in the low-elevation areas. At 5.0 GHz, the proposed antenna exhibits good broadside radiation and low sidelobe levels. The peak sidelobe levels (PSLs) in both planes are lower than -12.5 dB. The antenna also generates a broadside radiation pattern with a narrow main beam at 13.5 GHz. Due to the large inter-element spacing (about 1.85$\lambda _{\mathrm{{0}}}$, $\lambda _{\mathrm{{0}}}$ is the free-space wavelength at the frequency), the PSL of the array antenna is about -8.1 dB. Note that, no grating lobes emerge in the whole space. Besides, it can be found that the cross-polarization (XP) radiation is quite low in both low- and high-frequency bands, although the measured one is about 10 dB higher than the simulated one in the LF band.

Table 1 shows some comparisons between the proposed antenna and several published shared-aperture designs. It is observed that, due to the flexibility of the multi-antenna layout, the stacked designs reported in^20^ and^21^ can achieve broad impedance bandwidths in both operation bands. However, in order to avoid the block effect between multi-layer antennas, additional structures were required for the antenna design. Besides, the stacked designs may have higher profiles than others. In^2^ and^15^, the high-frequency substrate integrated waveguide (SIW) slot array structures were reused as radiation patch in the low-frequency band, which results in a high aperture reuse ratio. However, there exists only one low-frequency element in these designs. In this case, the peak gain in low-frequency band is limited. In^16^, by reusing the structure of high-frequency elements, high realized gains were obtained in both bands. However, the complex feeding network significantly increase the profile of the antenna. Compared with the above designs, the ratio between the numbers of high- and low-frequency elements (HLR) is significantly decreased by integrating 4 $\times$ 4 *C*-band hybrid ZOR patches and 2 $\times$ 2 *Ku*-band higher-order metasurface antennas. Besides, the presented design also has the advantages of high gain, low profile and high structure reuse ratio. Therefore, the proposed shared-aperture array is considered as a suitable candidate for future vehicular terminal applications.

**Table 1:** Performance comparison between several shared-aperture designs.

Reference	Mechanism	Impedance bandwidth	Peak gain(dBi)	Dimension	HLR	Reuse ratio
[2]	Co-planar design	3.43$\sim$3.52 GHz(2.6$\%$) $/$ 58.6$\sim$62.5 GHz(6.4$\%$)	7.3 $/$ 24	0.42$\lambda _{\mathrm{{0}}}\times$0.30$\lambda _{\mathrm{{0}}}$ $\times$0.02$\lambda _{\mathrm{{0}}}$	144	0.77
[15]	Shared-structure design	3.37$\sim$3.6 GHz(6.5$\%$) $/$ 24.3$\sim$27.8 GHz(13$\%$)	8.1 $/$ 18.7	0.74$\lambda _{\mathrm{{0}}}\times$0.86$\lambda _{\mathrm{{0}}}$ $\times$0.07$\lambda _{\mathrm{{0}}}$	16	0.875
[16]	Shared-structure design	5.4$\sim$5.9 GHz(8.8$\%$) $/$ 13.2$\sim$14.2 GHz(7.3$\%$)	12.9 $/$ 19.3	1.19$\lambda _{\mathrm{{0}}}\times$1.19$\lambda _{\mathrm{{0}}}$ $\times$0.21$\lambda _{\mathrm{{0}}}$	4	1
[20]	Stacked design	1.8$\sim$2.7 GHz(40$\%$) $/$ 3.3$\sim$3.8 GHz(14.1$\%$)	8.6$/$ 7.2	1.41$\lambda _{\mathrm{{0}}}\times$0.64$\lambda _{\mathrm{{0}}}$ $\times$0.21$\lambda _{\mathrm{{0}}}$	4	NA
[21]	Stacked design	2.16$\sim$2.82 GHz(26.5$\%$) $/$ 10.2$\sim$13.2 GHz(25.6$\%$)	7.5$/$ 13.1	0.91$\lambda _{\mathrm{{0}}}\times$0.67$\lambda _{\mathrm{{0}}}$ $\times$0.097$\lambda _{\mathrm{{0}}}$	4	1
This work	ZOR patch and MA	4.74$\sim$5.12 GHz(7.1$\%$) $/$ 13.0$\sim$14.0 GHz(7.4$\%$)	12.7 $/$ 17.2	1.37$\lambda _{\mathrm{{0}}}\times$1.37$\lambda _{\mathrm{{0}}}\times$0.043$\lambda _{\mathrm{{0}}}$	0.25	1

## Conclusion

This letter presents a low-profile dual-port shared-aperture array with a structure reuse ratio of 100$\%$. The antenna element is composed of a 2$\times$2 hybrid ZOR patch array and a higher-order MA. The evolution of the element is presented, and its reflection and radiation characteristic are analyzed. Afterward, the element is adopted to design an array with 4$\times$4 hybrid ZOR patch elements and 2$\times$2 MAs. Several split-ring shaped and L-shaped slots are etched to enhance the isolation between the two ports and enhance the reflection performance in the high-frequency band. The final array antenna is fabricated and measured. Measured results indicate that the antenna has peak gains of 12.7 and 17.2 dBi in the low- and high-frequency bands, respectively. Besides, the array antenna shows the low XP radiation and low sidelobe levels. Due to the high realized gain in *C* and *Ku* bands (4.74 $\sim$ 5.12 GHz $/$ 13.0 $\sim$ 14.0 GHz), the integrated design can be used as the transmitter in wireless systems for the vehicle-to-base station and vehicle-to-satellite communications.

## Data Availability

All Data has been included in study.
